# *N*-(4-Methyl­phen­yl)formamide

**DOI:** 10.1107/S1600536812024300

**Published:** 2012-05-31

**Authors:** Min-Min Zhao

**Affiliations:** a College of Chemistry and Chemical Engineering, Southeast University, Nanjing 210096, People’s Republic of China

## Abstract

In the title compound, C_8_H_9_NO, the amide group makes a dihedral of 32.35 (1)° with the benzene ring. In the crystal, pairs of strong N—H⋯O hydrogen bonds link the mol­ecules into inversion dimers. Weak C—H⋯O inter­actions further connect the mol­ecules into chains along the *a* axis.

## Related literature

For the structures and properties of related compounds, see: Tam *et al.* (2003 ▶); Omondi *et al.* (2005 ▶).
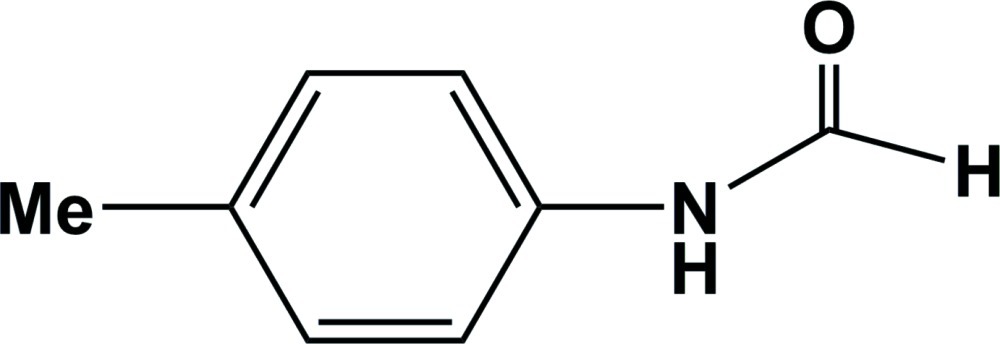


## Experimental

### 

#### Crystal data

C_8_H_9_NO
*M* *_r_* = 135.16
Triclinic, 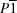

*a* = 6.5511 (11) Å
*b* = 6.9192 (12) Å
*c* = 8.0265 (17) Å
α = 93.730 (1)°
β = 102.780 (1)°
γ = 91.769 (1)°
*V* = 353.68 (11) Å^3^
*Z* = 2
Mo *K*α radiation
μ = 0.09 mm^−1^
*T* = 153 K
0.10 × 0.05 × 0.05 mm

#### Data collection

Rigaku Mercury2 diffractometer
Absorption correction: multi-scan (*CrystalClear*; Rigaku, 2005 ▶) *T*_min_ = 0.910, *T*_max_ = 1.000
2597 measured reflections
1570 independent reflections
943 reflections with *I* > 2σ(*I*)
*R*_int_ = 0.030

#### Refinement

*R*[*F* ^2^ > 2σ(*F* ^2^)] = 0.046
*wR*(*F* ^2^) = 0.127
*S* = 0.90
1570 reflections
92 parameters
H-atom parameters constrained
Δρ_max_ = 0.24 e Å^−3^
Δρ_min_ = −0.21 e Å^−3^

### 

Data collection: *CrystalClear* (Rigaku, 2005 ▶); cell refinement: *CrystalClear*; data reduction: *CrystalClear*; program(s) used to solve structure: *SHELXS97* (Sheldrick, 2008 ▶); program(s) used to refine structure: *SHELXL97* (Sheldrick, 2008 ▶); molecular graphics: *SHELXTL* (Sheldrick, 2008 ▶); software used to prepare material for publication: *SHELXTL*.

## Supplementary Material

Crystal structure: contains datablock(s) I, global. DOI: 10.1107/S1600536812024300/pv2549sup1.cif

Structure factors: contains datablock(s) I. DOI: 10.1107/S1600536812024300/pv2549Isup2.hkl

Supplementary material file. DOI: 10.1107/S1600536812024300/pv2549Isup3.cml

Additional supplementary materials:  crystallographic information; 3D view; checkCIF report

## Figures and Tables

**Table 1:** Hydrogen-bond geometry (Å, °)

*D*—H⋯*A*	*D*—H	H⋯*A*	*D*⋯*A*	*D*—H⋯*A*
N1—H1*B*⋯O1^i^	0.86	1.99	2.849 (2)	172
C7—H7*A*⋯O1^ii^	0.93	2.63	3.546 (2)	171

Symmetry codes: (i) 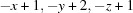; (ii) 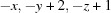.

**Fig. 1.**
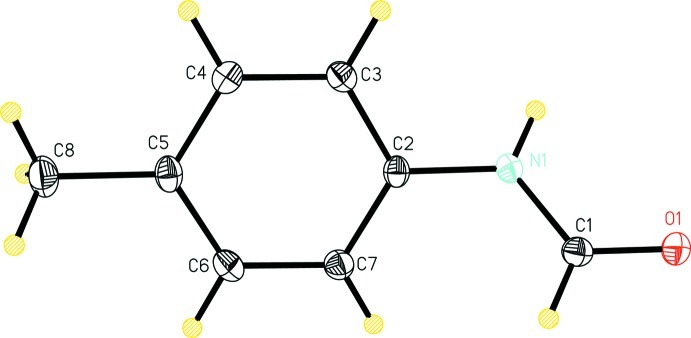
The molecular structure of the title compound with the atom numbering scheme. Displacement ellipsoids are drawn at the 50% probability level. H atoms are presented as small spheres of arbitrary radius.

**Fig. 2.**
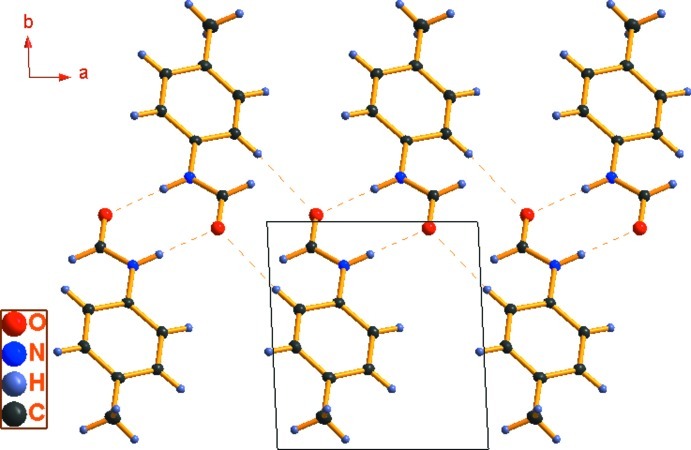
A view of the N—H···O and C—-H···O hydrogen bonds (dotted lines) in the crystal structure of the title compound. H atoms non-participating in hydrogen-bonding were omitted for clarity.

## supplementary crystallographic information

### Comment

N-(4-Chlorophenyl)formamide and N-(2,6-dichlorophenyl)formamide exhibit phase transitions under different thermal conditions from disordered model to ordered model (Tam *et al.*, 2003; Omondi *et al.*, 2005). Therefore, with the purpose of obtaining phase transition crystals of organic compounds, various similar organic molecules have been studied. The title compound has been synthesized to determine its crystal structure and dielectric properties. In this article, the synthesis and crystal structure of the title compound are reported.

In the title compound (Fig. 1), the amide group (O1/N1/C1) makes a dihedral of 32.35 (1)° with the benzene ring (C2–C7). In the crystal structure, the H atom bonded to the N atom is involved in a strong intermolecular N1—H1B···O1 hydrogen bond. In addition, weak C7—H7A···O1 further stabilize the crystal structure. These H-bonding interactions connect the molecules into a 1D chain along the *a*-axis (Fig. 2 and Table 1). The bond lengths and bond angles in the title molecule agree very well with the corresponding bond distances and bond angles reported in closely related compounds (Tam *et al.*, 2003; Omondi *et al.*, 2005)

### Experimental

A mixture of formic acid (30 mmol), 4-toluidine (10 mmol), H_2_SO_4_ (0.5 ml, molar concentration 98%) and ethanol (50 mL) in a 100 ml flask was stirred at 333 K for 10 h. Colourless crystals suitable for X-ray diffraction were obtained by slow evaporation of the solution.

### Refinement

All H atoms were positioned geometrically and refined using a riding model, with distances N—H = 0.86 Å and C—H = 0.93 and 0.96 Å, for aryl and methyl H-atoms, respectively. The *U*_iso_(H) were allowed at 1.5*U*_eq_(C methyl) or 1.2*U*_eq_(N/C non-methyl).

### Crystal data

**Table:**

C_8_H_9_NO	*Z* = 2
*M**_r_* = 135.16	*F*(000) = 144
Triclinic, *P*1	*D*_x_ = 1.269 Mg m^−^^3^
Hall symbol: -P 1	Mo *K*α radiation, λ = 0.71073 Å
*a* = 6.5511 (11) Å	Cell parameters from 1570 reflections
*b* = 6.9192 (12) Å	θ = 3.6–27.5°
*c* = 8.0265 (17) Å	µ = 0.09 mm^−^^1^
α = 93.730 (1)°	*T* = 153 K
β = 102.780 (1)°	Block, colorless
γ = 91.769 (1)°	0.10 × 0.05 × 0.05 mm
*V* = 353.68 (11) Å^3^	

### Data collection

**Table:**

Rigaku Mercury2 diffractometer	1570 independent reflections
Radiation source: fine-focus sealed tube	943 reflections with *I* > 2σ(*I*)
Graphite monochromator	*R*_int_ = 0.030
Detector resolution: 13.6612 pixels mm^-1^	θ_max_ = 27.5°, θ_min_ = 3.6°
CCD profile fitting scans	*h* = −8→8
Absorption correction: multi-scan (*CrystalClear*; Rigaku, 2005)	*k* = −7→8
*T*_min_ = 0.910, *T*_max_ = 1.000	*l* = −10→10
2597 measured reflections	

### Refinement

**Table:**

Refinement on *F*^2^	Primary atom site location: structure-invariant direct methods
Least-squares matrix: full	Secondary atom site location: difference Fourier map
*R*[*F*^2^ > 2σ(*F*^2^)] = 0.046	Hydrogen site location: inferred from neighbouring sites
*wR*(*F*^2^) = 0.127	H-atom parameters constrained
*S* = 0.90	*w* = 1/[σ^2^(*F*_o_^2^) + (0.0693*P*)^2^] where *P* = (*F*_o_^2^ + 2*F*_c_^2^)/3
1570 reflections	(Δ/σ)_max_ < 0.001
92 parameters	Δρ_max_ = 0.24 e Å^−^^3^
0 restraints	Δρ_min_ = −0.21 e Å^−^^3^

### Special details

**Table:**

Geometry. All esds (except the esd in the dihedral angle between two l.s. planes) are estimated using the full covariance matrix. The cell esds are taken into account individually in the estimation of esds in distances, angles and torsion angles; correlations between esds in cell parameters are only used when they are defined by crystal symmetry. An approximate (isotropic) treatment of cell esds is used for estimating esds involving l.s. planes.
Refinement. Refinement of F^2^ against ALL reflections. The weighted R-factor wR and goodness of fit S are based on F^2^, conventional R-factors R are based on F, with F set to zero for negative F^2^. The threshold expression of F^2^ > 2sigma(F^2^) is used only for calculating R-factors(gt) etc. and is not relevant to the choice of reflections for refinement. R-factors based on F^2^ are statistically about twice as large as those based on F, and R- factors based on ALL data will be even larger.

### Fractional atomic coordinates and isotropic or equivalent isotropic displacement parameters (Å^2^)

**Table:**

	*x*	*y*	*z*	*U*_iso_*/*U*_eq_	
O1	0.23436 (18)	1.02875 (15)	0.39355 (14)	0.0308 (3)	
N1	0.3558 (2)	0.80633 (18)	0.58427 (17)	0.0232 (3)	
H1B	0.4814	0.8558	0.6014	0.028*	
C7	0.1344 (3)	0.6126 (2)	0.7268 (2)	0.0247 (4)	
H7A	0.0295	0.7011	0.7049	0.030*	
C3	0.4782 (2)	0.5127 (2)	0.7131 (2)	0.0238 (4)	
H3A	0.6044	0.5333	0.6805	0.029*	
C2	0.3215 (2)	0.6435 (2)	0.67500 (19)	0.0210 (4)	
C4	0.4474 (3)	0.3510 (2)	0.7997 (2)	0.0249 (4)	
H4A	0.5542	0.2648	0.8251	0.030*	
C1	0.2053 (3)	0.8876 (2)	0.4739 (2)	0.0251 (4)	
H1A	0.0695	0.8344	0.4562	0.030*	
C6	0.1060 (3)	0.4493 (2)	0.8112 (2)	0.0261 (4)	
H6A	−0.0204	0.4285	0.8434	0.031*	
C5	0.2602 (3)	0.3151 (2)	0.8494 (2)	0.0251 (4)	
C8	0.2248 (3)	0.1390 (2)	0.9420 (2)	0.0359 (5)	
H8A	0.0850	0.0854	0.8973	0.054*	
H8B	0.2434	0.1753	1.0620	0.054*	
H8C	0.3236	0.0439	0.9255	0.054*	

### Atomic displacement parameters (Å^2^)

**Table:**

	*U*^11^	*U*^22^	*U*^33^	*U*^12^	*U*^13^	*U*^23^
O1	0.0283 (7)	0.0289 (7)	0.0364 (7)	0.0013 (5)	0.0065 (5)	0.0132 (6)
N1	0.0220 (7)	0.0227 (7)	0.0251 (7)	−0.0013 (5)	0.0053 (6)	0.0054 (6)
C7	0.0244 (9)	0.0237 (8)	0.0267 (9)	0.0018 (7)	0.0075 (7)	0.0018 (7)
C3	0.0231 (8)	0.0263 (9)	0.0226 (8)	−0.0012 (7)	0.0066 (7)	0.0017 (7)
C2	0.0258 (9)	0.0177 (8)	0.0189 (8)	−0.0014 (6)	0.0043 (6)	0.0011 (6)
C4	0.0268 (9)	0.0234 (9)	0.0238 (9)	0.0030 (7)	0.0039 (7)	0.0027 (7)
C1	0.0226 (9)	0.0255 (9)	0.0279 (9)	0.0018 (7)	0.0066 (7)	0.0029 (7)
C6	0.0251 (9)	0.0295 (9)	0.0248 (9)	−0.0042 (7)	0.0087 (7)	0.0013 (7)
C5	0.0322 (10)	0.0215 (8)	0.0202 (8)	−0.0045 (7)	0.0041 (7)	0.0015 (7)
C8	0.0412 (12)	0.0317 (10)	0.0355 (11)	−0.0041 (8)	0.0087 (9)	0.0111 (8)

### Geometric parameters (Å, º)

**Table:**

O1—C1	1.2369 (18)	C4—C5	1.391 (2)
N1—C1	1.3364 (19)	C4—H4A	0.9300
N1—C2	1.4189 (19)	C1—H1A	0.9300
N1—H1B	0.8600	C6—C5	1.391 (2)
C7—C6	1.383 (2)	C6—H6A	0.9300
C7—C2	1.393 (2)	C5—C8	1.506 (2)
C7—H7A	0.9300	C8—H8A	0.9600
C3—C2	1.387 (2)	C8—H8B	0.9600
C3—C4	1.387 (2)	C8—H8C	0.9600
C3—H3A	0.9300		
			
C1—N1—C2	124.15 (14)	O1—C1—N1	124.50 (15)
C1—N1—H1B	117.9	O1—C1—H1A	117.8
C2—N1—H1B	117.9	N1—C1—H1A	117.8
C6—C7—C2	119.50 (15)	C7—C6—C5	122.15 (15)
C6—C7—H7A	120.3	C7—C6—H6A	118.9
C2—C7—H7A	120.3	C5—C6—H6A	118.9
C2—C3—C4	120.16 (15)	C6—C5—C4	117.38 (14)
C2—C3—H3A	119.9	C6—C5—C8	120.91 (15)
C4—C3—H3A	119.9	C4—C5—C8	121.71 (15)
C3—C2—C7	119.37 (14)	C5—C8—H8A	109.5
C3—C2—N1	119.07 (14)	C5—C8—H8B	109.5
C7—C2—N1	121.56 (14)	H8A—C8—H8B	109.5
C3—C4—C5	121.42 (15)	C5—C8—H8C	109.5
C3—C4—H4A	119.3	H8A—C8—H8C	109.5
C5—C4—H4A	119.3	H8B—C8—H8C	109.5

### Hydrogen-bond geometry (Å, º)

**Table:**

*D*—H···*A*	*D*—H	H···*A*	*D*···*A*	*D*—H···*A*
N1—H1*B*···O1^i^	0.86	1.99	2.849 (2)	172
C7—H7*A*···O1^ii^	0.93	2.63	3.546 (2)	171

Symmetry codes: (i) −*x*+1, −*y*+2, −*z*+1; (ii) −*x*, −*y*+2, −*z*+1.
